# Association of *Anaplasma marginale* Strain Superinfection with Infection Prevalence within Tropical Regions

**DOI:** 10.1371/journal.pone.0120748

**Published:** 2015-03-20

**Authors:** Elizabeth J. Castañeda-Ortiz, Massaro W. Ueti, Minerva Camacho-Nuez, Juan J. Mosqueda, Michelle R. Mousel, Wendell C. Johnson, Guy H. Palmer

**Affiliations:** 1 Posgrado en Ciencias Genomicas, Universidad Autónoma de la Ciudad de México, México D.F., México; 2 Animal Disease Research Unit, USDA-ARS, Pullman, Washington, United States of America; 3 Department of Veterinary Microbiology and Pathology, Washington State University, Pullman, Washington, United States of America; 4 Universidad Autónoma de Querétaro, Campus Juriquilla, Juriquilla, México; 5 Paul G. Allen School for Global Animal Health, Washington State University, Pullman, Washington, United States of America; Kansas State University, UNITED STATES

## Abstract

Strain superinfection occurs when a second strain infects a host already infected with and having mounted an immune response to a primary strain. The incidence of superinfection with *Anaplasma marginale*, a tick-borne rickettsial pathogen of domestic and wild ruminants, has been shown to be higher in tropical versus temperate regions. This has been attributed to the higher prevalence of infection, with consequent immunity against primary strains and thus greater selective pressure for superinfection with antigenically distinct strains. However an alternative explanation would be the differences in the transmitting vector, *Dermacentor andersoni* in the studied temperate regions and *Rhipicephalus microplus* in the studied tropical regions. To address this question, we examined two tropical populations sharing the same vector, *R*. *microplus*, but with significantly different infection prevalence. Using two separate markers, *msp1α* (one allele per genome) and *msp2* (multiple alleles per genome), there were higher levels of multiple strain infections in the high infection prevalence as compared to the low prevalence population. The association of higher strain diversity with infection prevalence supports the hypothesis that high levels of infection prevalence and consequent population immunity is the predominant driver of strain superinfection.

## Introduction

Strain superinfection occurs when a second, genetically and antigenically distinct pathogen strain infects a host already infected with and having mounted an immune response to a primary strain of the same microbial species [1]. The ability of the second strain to evade the immune response generated by a primary strain has been shown to underlie superinfection for viral, bacterial, and protozoal pathogens, thus creating a selective pressure for antigenic divergence among strains [1–6]. At the host population level, high infection rates with a primary strain and consequent strain-specific immune responses would be expected to generate higher prevalence of superinfection as there would be few naïve hosts and new infections would require superinfection [7].

We and others have tested this model using *Anaplasma marginale*, a tick-borne rickettsial pathogen of domestic and wild ruminants. Superinfection is associated with strain-specific alleles encoding Major Surface Protein (Msp)-2, the immunodominant surface protein [4,8,9]. The second strain must encode at least one unique *msp2* allele with expression at the time of superinfection [4]. In tropical regions with high infection prevalence and consequent immunity, the rates of superinfection are significantly greater than in lower prevalence temperate regions [9,10]. That the superinfecting strains are genetically distinct has been demonstrated using both variable multi-locus analysis and detection of diverse sets of *msp2* alleles [9,10]. While these comparisons between tropical and temperate regions support the relationship between high infection prevalence and superinfection levels, there is an alternative explanation in that the transmitting vector in tropical regions, *Rhipicephalus microplus*, differs from that in the examined temperate regions, *Dermacentor andersoni* [11–13]. To determine whether the relationship between infection prevalence and strain superinfection also held in regions where transmission was mediated by the same vector, we identified two tropical regions with significantly different infection prevalence and determined the number of genotypically unique strains within infected animals using a strain-specific marker, *msp1α*, and the *msp2* repertoire [9,14,15]. Herein we report the results of these analyses and discuss the results in the context of strain superinfection.

## Materials and Methods

### Study sites and determination of infection prevalence

**Fig 1:**
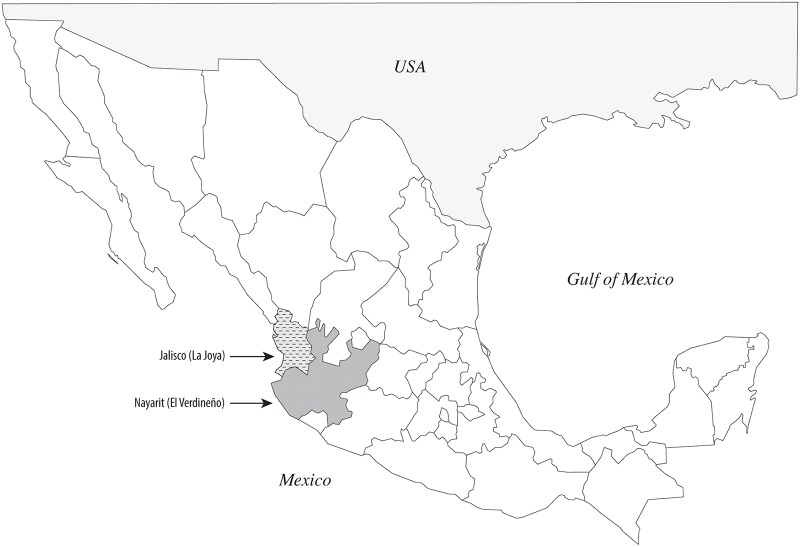
Study sites. A) Prevalence of infection as determined using serology and nested PCR and B) Geographic location of the herds in Mexico. The location of Santiago Ixcuintla, Nayarit is 21°48’N, 105°12’ W and Tapalpa, Jalisco is 19° 36’ N, 103° 36’ W.

Two cattle herds within tropical regions in Mexico were selected for the study; these herds, designated La Joya and El Verdineño, were located in the states of Jalisco and Nayarit, respectively (Fig. 1). The El Verdineño herd is located in the National Research Institute for Forestry, Agriculture and Livestock (INIFAP) station at Santiago Ixcuintla, Nayarit (21°48’N, 105°12’ W). The La Joya herd is located near the INIFAP station at Tapalpa, Jalisco (19°36’N, 103°36’W). *Rhipicephalus microplus* is present in both sites, consistent with its broad distribution in tropical Mexico. Blood samples were collected within the same month from approximately 80 animals per site with matching for age (8–12 months). None of the animals had been vaccinated against *A*. *marginale* nor treated with antibiotics to clear the infection. Infection prevalence was determined using the Msp5 competitive enzyme linked immunosorbent assay (CELISA) according to the manufacturer’s guidelines (VMRD). From the subsets of seropositive animals, genomic DNA was then extracted (Qiagen) and *msp5* PCR used to confirm *A*. *marginale* bacteremic animals [11].

### Strain identification using *msp1α* genotyping

*A*. *marginale* strains were identified by amplifying, cloning, and sequencing *msp1α*, a single copy chromosomal gene that is present in all examined strains but differs among strains in the number and sequence of nucleotide repeats [14,16,17]. To amplify the *msp1α* tandem repeat region, primers from a flanking conserved region present in all examined *A*. *marginale* were designed. The primer sets (forward 5′-GTG CTT ATG GCA GAC ATT TCC- 3’ and reverse 5’- CTC AAC ACT CGC AAC CTT GG- 3’) were used with PCR conditions of 94°C for 5 min; 30 cycles of 94°C for 30 sec, 57°C for 30 sec, 72°C for 35 sec, and a final extension of 72°C for 7 min. An internal primer msp1αNF (5’-CGC ATT ACA CGT TCC GTA TG-3’) was used with the reverse primer from the first reaction to increase the sensitivity for detecting *msp1α* at low levels of bacteremia. For the semi-nested PCR, the conditions were: 94°C for 5 min, 30 cycles of 94°C for 35 sec, 65°C for 58 sec, 72°C for 30 sec and a final extension of 72°C for 10 min. Amplicons were cloned into pCR4-TOPO-TA (Invitrogen) and plasmid DNA from at least five clones of each sample was extracted with Wizard plus SV minipreps (Promega). The presence of the insert was confirmed by *Eco*RI digestion and sequenced in both directions with a Big Dye kit and an ABI 3100 Prism automated sequencer (Applied Biosystems). The sequences were analyzed using the program BIOEDIT [18] to identify genotypes.

### Anaplasma marginale *msp2* allelic repertoire

The *msp2* allelic repertoire was determined for a subset of samples from each site by amplifying genomic DNA using primer sets *msp2*F (5′- CTG CTT TAG GTA AGA TGA CTA AGR GTG ARG C- 3’) in combination with each of five reverse primer sets as previously described [9]. The amplicons were cloned into pCR4-TOPO-TA (Invitrogen) and plasmid DNA from 96 bacterial clones of each sample was extracted with Wizard plus SV minipreps (Promega). All inserts were sequenced in both directions as described above. The sequences were edited using BioEdit software and analyzed with HyPhy 2.0 [19].

### Ethics statement

The protocol for animal handling and venipuncture was approved by the Comité Interno de Desarrollo Científico of INIFAP and the Washington State University Institutional Animal Care and Use Committee (protocol 04–326). Cattle were manually restrained (<5 minutes) and blood samples were obtained by jugular venipuncture (<0.0005% blood volume; one sampling per animal). The El Verdineño herd belongs to and was sampled with approval of the National Research Institute for Forestry, Agriculture and Livestock (INIFAP). The La Joya herd is privately owned and was sampled with approval of the owner.

## Results

### Infection prevalence

The infection rate in La Joya, Jalisco was 36% (30 positive/84 sampled) while that in El Verdineño, Nayarit was 100% (82/82). The difference was statistically significant (Fisher’s Exact Test p<0.0001).

### Strain identification using msp1α genotyping

Blood from bacteremic (*msp5* PCR positive) animals infected with *A*. *marginale* was further analyzed to determine if these animals were infected with a single or multiple *A*. *marginale* genotypes. Based on cloning and sequencing of *msp1α* (Table 1, S1 Table, S2 Table) there was a statistically significant difference (Fisher’s Exact Test, p = 0.009) in the proportion of superinfected animals between the high prevalence El Verdineño herd (86%; 37/43) and the lower prevalence La Joya herd (50%; 7/14). Using *msp1α* sequence as a genotypic marker for individual strains, the mean number of strains per infected animal in the high prevalence El Verdineño herd was 3.5±2.1, significantly greater (p = 0.03, unpaired t-test) than the 2.1±1.4 strains in the lower prevalence La Joya herd. The majority of the *msp1α* repeat sequences had been previously reported in independent studies; all newly reported sequences (Table 2) were identified in at least two infected animals and the same sequence identified by independent amplification and sequencing in different laboratories by different individuals. The new *msp1α* sequences have been deposited in GenBank (accession numbers KF791969–KF792017); accession numbers for all *msp1α* repeat sequences in the El Verdineño and La Joya animals are provided in S3 Table.

**Table 1:** Number of unique *A. marginale msp1α* genotypes detected per infected animal^a^.

Number of unique genotypes	Number of animals	
	El Verdineño	La Joya
1	6	7
2	11	2
3	10	2
4	5	2
5	3	1
6	4	0
7	2	0
8	0	0
9	2	0

^a^Based on number and sequence of repeats

**Table 2:** Newly identified *A*. *marginale msp1*α genotypes detected in infected animals from El Verdineño and La Joya.

Genotype	Access #	Sequence of encoded repeats^a^
A	M32871	DDSSSASGQQQESSVSSQSE-ASTSSQLG
EV1	KF791980	T******S*******L***DQ********
EV2	KF791982	T**********G*******GQ********
EV3	KF791981	A*****G************G*********
EV4	KF791977	T*****GD*******L*P*GQ********VG
EV5	KF791975	A*G*****———*****GQ********
EV6	KF791981	A*G*****———*L**GGQ********
EV7	KF791982	T**************L*P*GQ********VG
EV8	KF791990	T**********G*G*****GQ********
EV9	KF791996	AG****G********L*************G
EV10	KF791986	A*G***GD***********D*********
EV11	KF791978	A*******———*L*R*GQ********
EV12	KF791973	T*****GD***G*G*****GQ******S*
LJ1	KF791991	T**************L*P*GQ******S*
LJ2	KF791993	T*************A****D*********

^a^Comparison to the prototype *A*. *marginale* Florida strain Msp1a tandem repeat (A) with repeats found in El Verdineño (EV) and La Joya (LJ) infected animals. A star (*)indicates an identical amino acid and a dash indicates a deletion.

### *Msp2* repertoire

The *msp2* repertoire within an infected host is determined by both the coding alleles (<10 per strain) and the expressed copy (1 per bacterium but oligoclonal at a given timepoint in infection) [20,21]. Examination of experimentally superinfected animals reveals a marked and statistically significant increase in the repertoire as compared to animals infected with a single strain infected [9]. Using >3000 *msp2* sequences, ~300 to 700 sequences per individual animal, there was a statistically significant increase (p = 0.03, unpaired t-test) in the *msp2* repertoire in identified superinfected animals from El Verdineño as compared to single strain infected animals from La Joya. The mean number of *msp2* sequences in the selected El Verdineño cohort was 71±27 as compared to 10±2 in the La Joya cohort. The majority of the *msp2* sequences and the individual hypervariable microdomains have been previously reported [9]. Newly identified sequences are provided in S4 Table and have been deposited in GenBank (accession numbers KM388991-KM389141).

## Discussion

The present study supports the overall hypothesis that strain superinfection increases with higher levels of infection prevalence, providing increased selective pressure at the population level for transmission of genetically distinct strains [7,9]. This conclusion is based on three observations. First, there was a significant increase (p = 0.009) in the proportion of the population infected with ≥2 *msp1α* genotypes in the high prevalence El Verdineño herd as compared to La Joya. Second, the absolute number of genotypes per infected animal was significantly greater in the high prevalence herd (p = 0.03). The *msp1α* genotype has been shown to be a marker for individual strain identity; whole genome sequencing of strains initially identified by *msp1α* genotype has confirmed that they differ in multiple sites across the genome [10,21,22]. Third, independent analysis of the *msp2* repertoire in cohorts of infected animals revealed a statistically significant increase (p = 0.03) in the number of *msp2* sequences in infected animals from the high prevalence El Verdineño herd as compared to La Joya. The size of the *msp2* repertoire, derived from 5–10 chromosomal loci per strain and a single expression site locus [8,17,21], reflects not only infection with multiple strains, but also that the strains encode unique variants of the immunodominant surface proteins required for strain superinfection [4,8,9].

The results are consistent with two previous studies examining strain superinfection between high infection prevalence herds in tropical regions with low prevalence populations in temperate regions. Using analysis of the *msp2* repertoire, Ueti *et al*. demonstrated a statistically significant (p<0.0001) increase in the repertoire in infected animals in tropical regions as compared to both low prevalence temperate regions and experimental single strain infections [9]. The expansion of the repertoire was accompanied by an increase in the sequence diversity of the encoded Msp2 proteins and an increase in the number of unique combinations of hypervariable microdomains within the surface exposed Msp2 domain, both observations consistent with immune evasion during strain superinfection [9,23,24]. Using a multi-locus analysis independent of both *msp1α* and *msp*2, Vallejo-Esquerra *et al*. identified a significant increase (p = 0.0004) in superinfection in a high (>90%) prevalence tropical population (Veracruz, Mexico) as compared to temperate regions in the United States [10]. The detection of 3.3±1.2 strains in the Veracruz study using multi-locus typing [10] is similar to the detection of 3.5±2.1 unique *msp1α* genotypes in the present study; this concordance between high prevalence herds in different regions in the tropics and using independent samples and methods supports the relationship between superinfection and prevalence.

The prior studies comparing occurrence of superinfection with infection prevalence were potentially confounded by differences in the tick vector between the studied temperate regions, where *Dermacentor andersoni* is the vector and *R*. *microplus* is not present, and the tropical regions, where *R*. *microplus* is the vector and *D*. *andersoni* is absent [12,13]. Conceivably, *R*. *microplus* could be responsible for enhanced transmission of a second strain due to higher infection rates (percentage of fed ticks that become infected) or levels within the infected ticks that provided a transmission advantage. Direct comparison of infection rates were not significantly different using *R*. *microplus* versus *D*. *andersoni* fed on animals infected with either a temperate or tropical strain [11]. However, the tropical strain, isolated in Puerto Rico, replicated in the salivary glands of *R*. *microplus* at the time of transmission to levels over 10^6^ organisms/salivary gland as compared to the temperate strain levels in *D*. *andersoni* of approximately 10^3.5^ organisms/salivary gland [11]. The present study resolved the question as to whether the association between superinfection and prevalence was maintained under conditions where the same vector tick species was responsible for transmission. *R*. *microplus* is present in both the La Joya and El Verdineño study sites, consistent with prior mapping studies, and no other known tick vectors for *A*. *marginale* are present in these regions [12, 25]. Whether differences exist at the sub-species level, e.g., genetically distinct populations of *R*. *microplus*, in the capacity to transmit *A*. *marginale* and if these may underlie differences in infection prevalence within tropical regions is unknown. Scoles et al. reported that geographically separated populations of the temperate vector tick *Dermacentor andersoni* differ in susceptibility to *A*. *marginale* at the level of the midgut [26], however whether this affects transmission efficiency or incidence of superinfection remains unresolved.

The present findings support that infection prevalence, associated with immunity against a primary strain and then selecting for superinfection with additional genetically and antigenically distinct strains [7], is the unifying link with the occurrence of superinfection. Whether strain superinfection occurs as a linear function of primary strain infection prevalence within the host population or, alternatively, reflects threshold functions in primary strain infection prevalence and immunity is unknown. Inclusion of additional herds with differing levels of infection prevalence and examining occurrence of superinfection relative to primary infection over time are approaches that can be used to test these hypotheses and further advance understanding of strain superinfection.

## Supporting Information

S1 Table *A*. *marginale msp1*α genotypes present in singly and superinfected cattle in El Verdineño (Nayarit). The unique variable repeat sequence is designated by a letter, number, or alphanumeric combination. The sequences have been deposited in GenBank and the accession numbers are provided in S3 Table. The *msp1*α genotype is designated by the number and order of the repeats, listed 5’ to 3’.
(PDF)

S2 Table *A*. *marginale* msp1α genotypes present in singly and superinfected cattle in La Joya (Jalisco). The unique variable repeat sequence is designated by a letter, number, or alphanumeric combination. The sequences have been deposited in GenBank and the accession numbers are provided in S3 Table. The *msp1*α genotype is designated by the number and order of the repeats, listed 5’ to 3’.
(PDF)

S3 Table *A*. *marginale msp1α* repeat sequences in the current study. The repeat designation, strain name and location of first reported isolation, and GenBank accession number.
(PDF)

S4 Table Unique *A*. *marginale* Msp2 hypervariable regions identified in the current study. The animal number, GenBank accession number, and encoded hypervariable sequences.
(PDF)
