# Correction: Association of *Anaplasma marginale* Strain Superinfection with Infection Prevalence within Tropical Regions

**DOI:** 10.1371/journal.pone.0129415

**Published:** 2015-05-28

**Authors:** Elizabeth J. Castañeda-Ortiz, Massaro W. Ueti, Minerva Camacho-Nuez, Juan J. Mosqueda, Michelle R. Mousel, Wendell C. Johnson, Guy H. Palmer

In Fig 1, Nayarit and Jalisco were inadvertently reversed. All other references and data for the study sites are correct. Please view the correct Fig 1 here.

**Fig 1:**
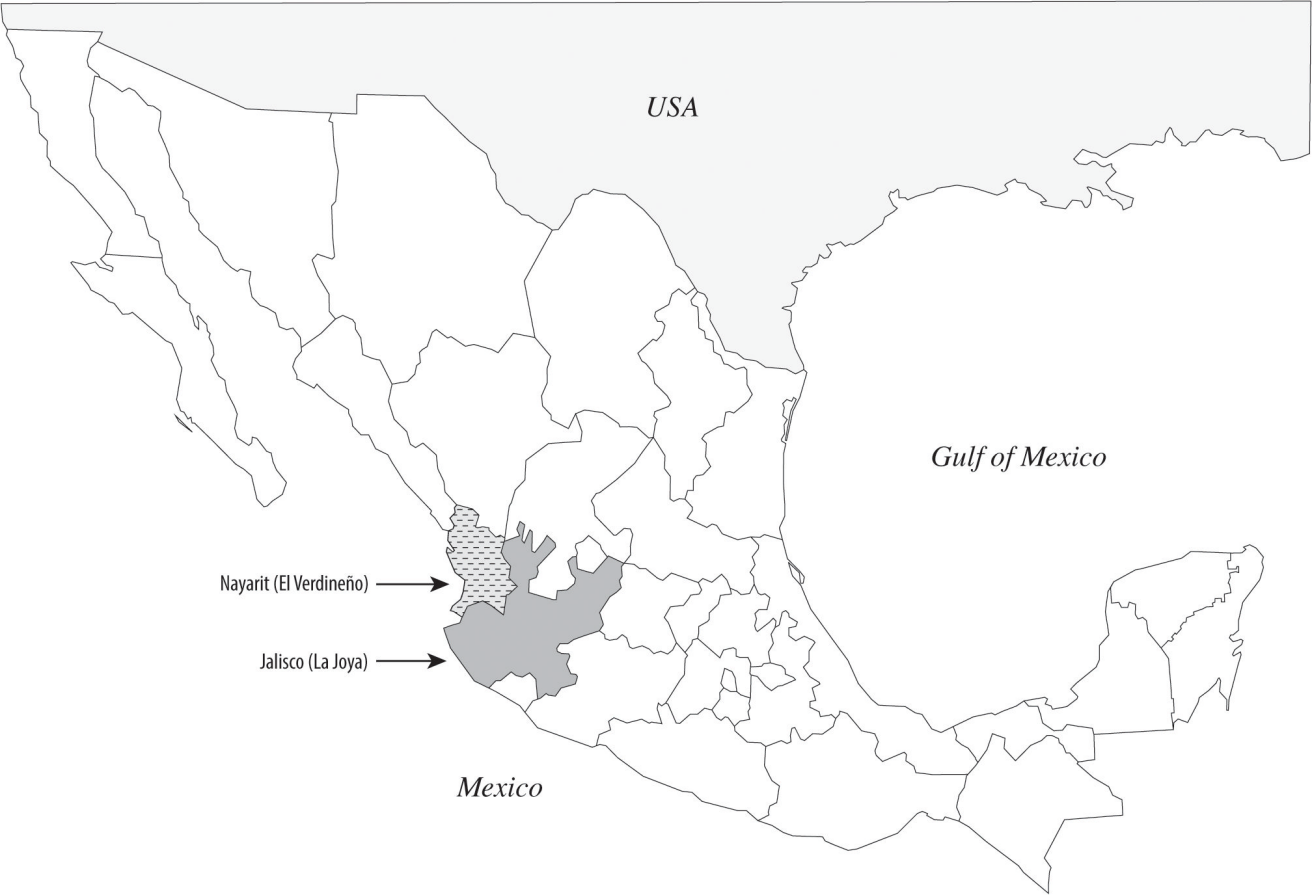
Study sites. A) Prevalence of infection as determined using serology and nested PCR and B) Geographic location of the herds in Mexico. The location of Santiago Ixcuintla, Nayarit is 21°48’N, 105°12’ W and Tapalpa, Jalisco is 19° 36’ N, 103° 36’ W.
