# Supplementary material for: Association of Anaplasma marginale Strain Superinfection with Infection Prevalence within Tropical Regions
Source: PLoS One. 2015 Mar 20;10(3):e0120748. doi: 10.1371/journal.pone.0120748 (PMC4368111; doi:10.1371/journal.pone.0120748)
Supplement: S1 Table — The unique variable repeat sequence is designated by a letter, number, or alphanumeric combination. The sequences have been deposited in GenBank and the accession numbers are provided in S3 Table. The msp1α genotype is designated by the number and order of the repeats, listed 5’ to 3’. (PDF) [file pone.0120748.s001.pdf]

S1 Table: *A. marginale msp1*α genotypes present in singly and superinfected cattle in El Verdineño (Nayarit)

	Number of unique *msp1α* genotypes detected and sequenced[a]								
Animal #	1	2	3	4	5	6	7	8	9
4511	EV12,10,15								
8504	28,29,74,29,M,F								
6032	Isr4,9,10,10,11,9								
3615	60,62,62,61,62,62								
7557	28,29,74,29,M,F								
6514	EV4,62,62,EV11,9,62,62,EV11,9								
2085	EV2,EV7,EV7,EV7,EV7	EV1,βββΓ							
2087	28,29,74,29,M,F	EV9,13,13							
3579	13,13,13,13,13,13	T,Q,C,N,B,C,EV8							
3607	Isr4,9,10,Isr3,9,10,11,10	αββΓ							
3611	13,13,13,13	EV12,10,15							
3616	13,13,13,13,13,13	T,Q,C,N,B,C,EV8							
4525	αβββΓΓ	αββΓ							
4536	T,Q,C,N,B,C,EV8	αβββΓΓ							
4548	αββΓ	T,C							
4592	EV10,tc63_3_s06 EB	13,13,13,13							
8519	28,29,74,29,M,F	EV4,62,EV11,9							
043	EV4,62,62,EV11,9	T,B,C,N,B,C,F	13,13,13,13						
049	28,29,74,29,M,F	13,13,13,13	EV1ββΓ						
1545	60,61,62,62,62,61	13,13,13,13	13,13						
2089	EV4,62,62,62,62,EV11,9	EV3,EV7,ββ,EV6	28,29,74,29,M,F						
3533	13,13,13,13,13,13	T,Q,C,N,B,C,EV8	T,C,C						
4511	13,13,13,13	EV12,10,15	αβββΓΓ						
4519	13,13,13,13,13,13	T,B,C,N,B,C,F	EV1,βββΓ						
8536	28,29,74,29,M,F	EV12,10,15	αββΓ						
1549	EV9,13,13,13,13	EV12,10,15,15	τ,10,15,15						
1562	4,9,3,9,11,9	13,13,13,13,13,13	T,B,C,N,B,C,F						
3571	EV5,62,62,61,62,61	4,9,10,10,11,9	Isr4,Ita5,61	τ,10,15,15					
3592	Isr4,9,10,Isr3,9,10,10,9	EV9,13,13,13,13	EV12,10,15	4,9,10,11,M					
3518	EV12,10,15,15	τ,10,15,15	13,13,13,13	4,9,10,10,11,9					
3597	13,13,13,13,13,13	EV12,10,15	13,13,13,13,13	T,C					
1559	28,29,74,29,M,F	EV12,10,15,15	13,18	13,13,13					
1572	EV4,62,62,EV11,9	T,B,C,N,B,C,F	13,13,13,13	EV1,βββΓ	τ,10,15,15				
1538	13,13,13,13,13,13	T,Q,C,N,B,C,EV8	τ,10,15	4,9,10,3,9,10,11,9	EV12,10,15				
5552	αβββΓΓ	αβΓΓ	ββ,LJ1	αββΓ	αβΓ				
1546	28,29,EV5,29,M,F	T,B,C,N,B,C,F	EV1,EV2,EV2,9	EV3,EV7,ββ,EV6	EV12,10,15	αββΓ			
3600	EV2,EV7,EV7,EV7,EV7	EV12,10,15	T,B,C,N,B,C,F	13,13,13,13	αβββΓΓ	αββΓ			
4538	60,61,62,62,62,61	Isr4,9,10,10,11,9	EV10, tc63_3_s06 EB	28,29,74,62,61	28,29,74,29,M,F	EV12,10,15,15			
5591	EV12,10,15,15	EV3,β,β,EV6	EV3,EV7,β,EV6	EV3,ββ,EV6	αββββΓ	αβββΓ			
5585	EV12,10,15,15	T,C,C,B,C	EV12,10,15	EV9,13,ΓΓ	C,C,C	αββ	T,C		
1527	T,Q,C,N,B,C,EV8	EV4,62,62,EV11,9	T,B,C,N,B,C,F	T,Q,C,N,B,C,F	13,F,B,C,EV8	αββ,LJ1,Γ	αβββΓΓ		
3603	EV2,EV7,EV7,EV7,EV7	EV5,62,61,61,62	EV4,62,62,EV11,9	α,EV7,EV7,EV7	60,62,62,61,62	EV12,10,15,15	EV12,10,15	αββΓ	T,C
4517	EV2,EV7,EV7,EV7,EV7	EV11,tc63_3_s06,EB	28,29,EV5,29,M,F	T,B,C,N,B,C,F	13,13,13,13	EV1,βββΓ	EV12,10,15	αβββΓ	αβΓΓ

[a] The unique variable repeat sequence is designated by a letter, number, or alphanumeric combination. The sequences have been deposited in GenBank and the accession numbers are provided in S3 Table. The *msp1*α genotype is designated by the number and order of the repeats, listed 5’ to 3’.
