# Supplementary material for: Association of Anaplasma marginale Strain Superinfection with Infection Prevalence within Tropical Regions
Source: PLoS One. 2015 Mar 20;10(3):e0120748. doi: 10.1371/journal.pone.0120748 (PMC4368111; doi:10.1371/journal.pone.0120748)
Supplement: S2 Table — The unique variable repeat sequence is designated by a letter, number, or alphanumeric combination. The sequences have been deposited in GenBank and the accession numbers are provided in S3 Table. The msp1α genotype is designated by the number and order of the repeats, listed 5’ to 3’. (PDF) [file pone.0120748.s002.pdf]

S2 Table: *A. marginale msp1*α genotypes present in singly and superinfected cattle in La Joya (Jalisco)

	Number of unique *msp1α* genotypes detected and sequenced[a]				
Animal	1	2	3	4	5
01A	12,13,13,14				
32B	τ,57				
40N	12,13				
52N	LJ2,13,18				
54N	αβββΓΓ				
68N	αββ15				
90N	αββ,LJ1				
10B	αββΓΓ	αβββΓΓ			
09N	T,Q,C,N,B,C,F	EV4,C,EV8			
02V	LJ2,LJ2,13,18	LJ2,13,18	18		
05A	EV2,EV7,EV7,EV7,EV7	T,Q,C,N,B,C,EV8	T,B,C,N,B,C,F		
01N	α,EV6,β,LJ1	EV4,62,62,EV11,9	EV12,10,15,15	αββ,LJ1	
06B	EV4,62,62,EV11,9	LJ2,LJ2,13,18	LJ2,13,18	αβββΓΓ	
35B	EV2,EV7,EV7,EV7,EV7	EV3,EV7,ββ,EV6	LJ2,13,18	13,18	τ,57

[a] The unique variable repeat sequence is designated by a letter, number, or alphanumeric combination. The sequences have been deposited in GenBank and the accession numbers are provided in S3 Table. The *msp1*α genotype is designated by the number and order of the repeats, listed 5’ to 3’.
