# Supplementary material for: Association of Anaplasma marginale Strain Superinfection with Infection Prevalence within Tropical Regions
Source: PLoS One. 2015 Mar 20;10(3):e0120748. doi: 10.1371/journal.pone.0120748 (PMC4368111; doi:10.1371/journal.pone.0120748)
Supplement: S3 Table — The repeat designation, strain name and location of first reported isolation, and GenBank accession number. (PDF) [file pone.0120748.s003.pdf]

S3 Table: *A. marginale msp1α* repeat sequences in the current study

Repeat Designation	Strain Name and Location of First Report	Access #
B	Florida, USA	M32871
C	Washington, USA	M32869
E	Idaho, USA	M32868
F	Quitilipi, Argentina	DQ833270
M	Quitilipi, Argentina	DQ833270
N	Cushing, USA	AY127057
Q	Canadian bison, Canada	AY253141
T	Yucatan, Mexico	AF345871
EV1	El Verdineño, Mexico	KF791980
EV2	El Verdineño, Mexico	KF791982
EV3	El Verdineño, Mexico	KF791981
EV4	El Verdineño, Mexico	KF791977
EV5	El Verdineño, Mexico	KF791975
EV6	El Verdineño, Mexico	KF791981
EV7	El Verdineño, Mexico	KF791982
EV8	El Verdineño, Mexico	KF791990
EV9	El Verdineño, Mexico	KF791996
EV10	El Verdineño, Mexico	KF791986
EV11	El Verdineño, Mexico	KF791978
EV12	El Verdineño, Mexico	KF791973
Isr3	Israel tailed, Israel	AY295077
Isr4	Israel non-tailed, Israel	AF352559
Ita5	Italy 32, Italy	AY702928
tc63_3_s06	Yaracuy 1585, Venezuela	AFJ00024
LJ1	La Joya, Mexico	KF791991
LJ2	La Joya, Mexico	KF791993
α	Morelos, Mexico	AF345869
β	Morelos, Mexico	AF345869
Γ	Morelos, Mexico	AF345869
τ	Parana, Brazil	AY998121
3	SA302, South Africa	DQ813548
4	Aguascalientes, Mexico	DQ501243
9	Aguascalientes, Mexico	DQ501243
10	Aguascalientes, Mexico	DQ501243
11	Aguascalientes, Mexico	DQ501243
12	Puente de Ixtla, Mexico	DQ501242
13	Puente de Ixtla, Mexico	DQ501242
14	Puente de Ixtla, Mexico	DQ501242
15	Parana, Brazil	AY998121
18	Chaco, Argentina	DQ833266
28	Quitilipi, Argentina	DQ833270
29	Quitilipi, Argentina	DQ833270
57	Tamaulipas 1, Mexico	EU283847
60	Tamaulipas 4, Mexico	EU283849
61	Tamaulipas 4, Mexico	EU283849
62	Tamaulipas 4, Mexico	EU283849
74	Tamaulipas 2, Mexico	JN564651
