# Supplementary material for: Association of Anaplasma marginale Strain Superinfection with Infection Prevalence within Tropical Regions
Source: PLoS One. 2015 Mar 20;10(3):e0120748. doi: 10.1371/journal.pone.0120748 (PMC4368111; doi:10.1371/journal.pone.0120748)
Supplement: S4 Table — The animal number, GenBank accession number, and encoded hypervariable sequences. (PDF) [file pone.0120748.s004.pdf]

S4 Table: Unique *A. marginale* Msp2 hypervariable regions identified in the current study

Animal	Accession	Encoded MSP2 hypervariable region
3611	KM388991	ATIENTTGSGDVSQKVCGKGTLVVAPTSVVRTPPMAPPRSVRCSLRVRHSNLLSGGGDSINTTG
3611	KM388992	ATIENTTGSGDVSQKVCGKGTTSGSTNQCGKNTTDGTTKISAVFTDEATLLSAAGDTINTTG
3611	KM388993	ATIENTTGSGDVSQKVCGKGTTSGSTNQCGKNTTDGTTKISAVFTEGTDRATLLSAAGDTIHTTG
3611	KM388994	ATIENTTGSGDVSQKVCGKGTTSGSTNQCGKNTTDGTTKISAVFTEGTDTATLLSAGGDTINTTG
3611	KM388995	ATIENTTGSGDVSQKVCGKGTTSGSTNQCGKNTTDGTTKISEVFTDEATLLSAAGDTINTTG
3611	KM388996	ATIENTTGSGDVSQKVCKGDSGTCGKNTADGNATTKISAVFTEGTEAISSMETATISLQG
3611	KM388997	ATIENTTGSGDVSQKVCKGDSGTCGKNTSGNAAQTNGKLSTVFSDEGTDAISSMETATSGATISTSG
3611	KM388998	ATIENTTGSGDVSQKVCKGDSGTCGKNTSGTAAQTNGKLSTVFRDEGTDAISSMETAPISLQG
3611	KM388999	ATIENTTGSGDVSQKVCKGDSGTCGKNTSGTAAQTNGKLSTVFRDEGTDAISSMETATISLQG
3611	KM389000	ATIENTTGSGDVSQKVCKGDSGTCSKNTSGTAAQTNGKLSTVFSDEGTDAISSMETATISVQG
3611	KM389001	ATIENTTGSGDVSQKVCKGTGTGGTIQCGKNTADGNATTKISAVFTEGTEAISSMETATISLQG
3611	KM389002	ATIENTTGSGDVSQKVCKGTGTGGTNQCGKNTADGNAPTKISAVFTEGTEAISSMETATISLQG
3611	KM389003	ATIENTTGSGDVSQKVCKGTGTGGTNQCGKNTADGNATTKISAVFTEGTEAISSMETATISLR
3611	KM389004	KAVEGATGGDKVSQNVCKGDSGTKCGVNATSGTAQTKISAVFTEGTDTATQLSADTNNVSTSG
3611	KM389005	KAVEGTTNGEKVSQNVCGKGEGSNGTKKCGTTDSTATTKISAVFTEDAAAQLSTMDNTTINTSG
3611	KM389006	KAVEGTTNGEKVSQNVCGKGEGSNGTKKCGTTDSTATTKISAVFTEDAAAQLSTMDNTTINTTG
3611	KM389007	KAVEGTTNGEKVSQNVCGKGEGSNGTKKCGTTDSTATTKISEVFTEGTDTLLSVEGNKDTINFQG
3611	KM389008	KAVEGTTNGEKVSQNVCGKGEGSNGTKKCGTTDSTATTKISEVFTEGTDTLLSVEGNKDTINLQG
3611	KM389009	KAVEGVTGGDELSKEVCKGENNKCGVNATSGTAAQTNGKLSTVFSDEGTDAISSMETATSGATISTSG
3611	KM389010	KAVEGVTGGDELSKKVCGKGTTSGNQCGKNTTDGTTKISAVFTEGTDTATLLSAAGDTINTTG
3611	KM389011	KAVEGVTGGDELSKKVCGKGTTSGNQCGVNATSGSTTKISAVFTDEATLLSAAGDTINTTG
3611	KM389012	KAVEGVTGGDELSKKVCKGENNKCGVNATSGSPTKISAVFTDEATLLSAAGDTINTTG
3611	KM389013	KAVEGVTGGDELSKKVCKGENNKCGVNATSGSTTKISAVFTDEATLLSAAGDSLNTTG
3611, 4511 and 8536	KM389014	KAVEGVTGGDELSKKVCKGENNKCGVNATSGSTTKISAVFTDEATLLSAAGDTINTTG

3611	KM389015	KAVEGVTGGDELSKKVCKGENNKCGVNATSGSTTKISAVFTEGTDTAALLSAAGDTINTTG
3611	KM389016	KAVEGVTGGDELSKKVCKGENNKCGVNATSGSTTKISAVFTGEATLLSAAGDTINTTG
3611	KM389017	KAVEGVTGGDELSKKVCKGENNKCGVNATSGTAAQTNGKLSTVFSDEGTDAISLMETATSGATISSSG
3611	KM389018	KAVEGVTGGDELSKKVCKGENNKCGVNATSGTAAQTNGKLSTVFSDEGTDAISLMETATSGATISTSG
3611	KM389019	KAVEGVTGGDELSKKVCKGENNKCGVNATSSIAAQTNGKLSTVFSDEGTDAISSMEISTSGATISTSG
3611	KM389020	KAVEGVTGGDELSKKVCKGENNKCGVNATSSTAAQTNGKLSTVFSDEGTDAISSMETATSGATISTSG
3611	KM389021	KAVEGVTGGDELSKKVCKGENNKCGVNTTSGTAAQTNGKLSTVFSDEGTDAISSMETATSGATISTSG
3611	KM389022	KAVEGVTGGDELSKKVCKGENNQCGKNTSGTTATTQRKISEVFTSDTETAQLSSDTANINTTG
3611	KM389023	KAVEGVTGGDELSKKVCKGENNQCGKNTSGTTATTQRKISEVFTSDTETAQLSSDTTNINTTG
3611	KM389024	KAVEGVTGGDELSKKVCKGTGTGGTNQCGVNAARGTTTKISAVFTDEATLLSADTNNVSTSG
3611	KM389025	KAVEGVTGGDELSKKVGKGENNKCGVNATSGSTTKISAVFTEEATLLSAAGDTINTTG
3611	KM389026	KAVEGVTGGDKVSQNVCKGDSGTKCGVNATSGTAQTKISAVFTEGTDAATQLSADTNNVSTSG
3611, 4511 and 02V	KM389027	KAVEGVTGGDKVSQNVCRGDSGTKCGVNATSGTAQTKISAVFTEGTDTATQLSADTNNVSTSG
3611	KM389028	KAVENATNGAKVSQNVCGKGTGTGSDGNTKKCGTNDGTTATQRKISEVFTDEATLLSAAGDTINTTG
3611, 68N and 02V	KM389029	KAVENATNGDKVSQNVCGKGTGTGSDGNTKKCGTNDGTTATQRKISEVFTDEATLLSAAGDTINTTG
3611	KM389030	KAVENATNGDKVSQNVCGKGTGTGSDGNTKKCGTNDGTTATTKISAVFTEDAAAQLSTMDNTTINTTG
3611	KM389031	KAVENATNGDKVSQNVGGKGTGTGSDGNTKKCGTNDGTTATQRKISEVFTDEATLLSAAGDTINTTG
3611	KM389032	KAVKGVTGGDKVSQNVCKGDSGTKCGVNATSGTAQTKISAVFTEGTDTATQLSADTNNVSTSG
3611	KM389033	NAIESATGITNGEKVSQKVCGNGTSGTGGPQCGKNSGDTNGSSTTQHKISAVFTDEATLLSAAGDTINTTG
3611	KM389034	NAIESATGTTHGEKVSQKVCKGDSGTKCGVNATSGTAQTKISAVFTEGTDTATQLSADTNNVSTSG
3611	KM389035	NAIESATGTTNGEKVSQKVCGEGTTSGSTNQCGKNTTDGTTKISAVFTEGTDTATLLSAAGDTINTTG
3611	KM389036	NAIESATGTTNGEKVSQKVCGNGTSGTGGTQCGKNSGDTNGSSTTQHKISAVFTEEATLLSAAGDTINTTG
3611	KM389037	NAIESATGTTNGEKVSQKVCGNGTSGTGGTQCGKNSGDTNGSSTTQHKISAVFTEGTDTATLLSAAGDTINTTG
3611	KM389038	NAIESATGTTNGEKVSQKVCGNGTSGTGGTQCGKNSGDTNGSSTTQHKISAVSTDEATLLSAAGDTINTTG

3611	KM389039	NAIESATGTTNGEKVSQKVCGNGTSGTGGTQCGKNSGDTNGSSTTQHKISGVFTDEATLLSAAGDTINTTG
3611	KM389040	NAIESATGTTNGEKVSQKVCGNGTSGTGGTQCGKNSGDTNGSSTTQHKMSAVFTDEATLLSAAGDTINTTG
3611	KM389041	NAIESATGTTNGEKVSQKVCKGDSGTKCGVNATSGTAQTKISAVFTEGTDTATQLSADTNNVSTPG
3611	KM389042	NAIESATGTTNGEKVSQKVCKGDSGTKCGVNATSGTTQTKISAVFTEGTDTATQLSADTNNVSTSG
3611	KM389043	NAIESATGTTNGEKVSQRVCGNGTSGTGGTQCGKNSGDTNGSSTTQHKISAVFTDEATLLSAAGDTINTTG
3611	KM389044	NAIESATGTTNGGKVSQKVCKGDSGTKCGVNATSGTAQTKISAVFTEGTDTATQLSADTNNVSTSG
3611	KM389045	NAIESATGTTSGDTVSKKVCGKGTGSTGTTCGKSADSQSTNSKLGTVFNAEGADTATQLSADTNNVSTSG
3611	KM389046	NAIESDTGTTNGEKVSQKVCGNGTSGTGGTQCGKNSGDTNGSSTTHHKISAVSTDEATLLSAAGDTINTTG
3611	KM389047	NAIESVTGTTNGQTVSQKVCANGTGSSGSNCGKSADSQSTNSKLGTVFNAEGADAISSMDTTTTSGASGTISLQG
3611	KM389048	NAIESVTGTTNGQTVSQKVCGNGTGSSGSNCGKSADSQSTNSKLGTVFNAEGADAISSMDTTTTSGASDTISLQG
3611 and 4511	KM389049	NAIESVTGTTNGQTVSQKVCGNGTGSSGSNCGKSADSQSTNSKLGTVFNAEGADAISSMDTTTTSGASGTISLQG
3611	KM389050	NAIESVTGTTNGQTVSQKVCGNGTGSSGSNCGKSADSQSTNSKLGTVFNAEGADAISSMETATISLQG
3611	KM389051	NAIESVTGTTNGQTVSQKVCGNGTGSSGSNCGKSADSQSTNSKLGTVFNAEGADTATQLSADTNNVSTSG
3611	KM389052	NAIESVTGTTNGQTVSQKVCGNGTGSSGSNCGKSADSQSTNSKLGTVFNGEGADAISSMDTTTTSGASGTISLQG
3611	KM389053	NAIESVTGTTNGQTVSQKVCGNGTGSSGTQCGKNSGDPNGSSPTQQKISAVFTSDTETAQLSTMENTSTTSGATI
3611 and 4511	KM389054	NAIESVTGTTNGQTVSQKVCGNGTGSSGTQCGKNSGDTNGSSTTQHKISAVFTSDTETAQLSTMENTSTTSGATIS
3611	KM389055	NAIESVTGTTNGQTVSQKVCGNGTSGTGGTQCGKNSGDTNGSSTTQHKISAVFTDEATLLSAAGDTINTTG
3611	KM389056	TTIENTPGSGDELSKKVCGKGDSGTKCGVNATSGTAQTNGKLSTVFNTDGAEAISSMETATISVQG
3611	KM389057	TTIENTTASGYELSKKVCGKGDSGTKCGVNATSGTAQTNGKLSTVFNTDGAEAISSMETATISVQG
3611	KM389058	TTIENTTGSADELSKKVCGKGDSGTKCGVNATSGTAQTNGKLSTVFNTDGAEAISSMETATISVQG
3611	KM389059	TTIENTTGSGDELSKKVCGKGDSGTKCGVNATSGTAQTNGKFSTVFNTDGAEAISSMETATISVQG
3611	KM389060	TTIENTTGSGDELSKKVCGKGDSGTKCGVNATSGTAQTNGKLSTVFHTDGAEAISSMETATISVQG
3611	KM389061	TTIENTTGSGDELSKKVCGKGDSGTKCGVNATSGTAQTNGKLSTVFSDEGTDAISSMETATISLQG
3611 and 4511	KM389062	TTVEAATNGQTVSQKVCGNGTGSSGSNCGKNTTDSTNNNGKITQAFTADSDTTLLSAESSNISTSG

8536	KM389063	ATIENTTGSGDELSKKVCGKGDSGTKCGVNATSGTAAQTNGKLSTVFSDEGTDAISSMETATISLQG
8536	KM389064	DAIESATGTTNGDPVGKNVCRGTGADNSGNNCGPNDGNATTKISTVFNNEGTEAISSMDTTADGTSTSISLQG
8536	KM389065	KAVEGVTGGDELSKKVCKGTGTDSSGNKCGTNDGNATTKISTVFNTEGTEAISSMDTTASGTSNTISLQG
8536	KM389066	KAVEGVTGGDELSKKVCKGTGTGGTNQCGVNAARGTTTKISAVFTDEGRLLSADTNNVSTSG
8536	KM389067	KAVEGVTGGDELSKKVCKGTGTGGTNQCGVNAASGTTAQTKISAVFTDEATLLSADTNNVSTSG
8536	KM389068	KAVEGVTGGDKMSQNVNKGDSGTKWGVNATSGTPQTNISAVFIEGTDTATQLSADTNNVSTSG
8536	KM389069	KAVEGVTGGDKVSQNVCKGDSGTKCGVNATSGTAQTKISVVFTEGTDTATQLSADTNNVSTSG
8536	KM389070	NAIESATATTNGDTVSKNVCKGDSGTCGKNTSGTATTKISAVFTDEATLLSADTNNVSTSG
8536	KM389071	NAIESATGTSNGDTVSKNVCKGTGTSDTTNQCGTSTAGATTKISAVFTEDAAAQLSTMDTATSSTSTGTISLQG
8536	KM389072	NAIESATGTTNGDTVSKNVCKDDSGTCGKNTSGTATTKISAVFTEDAAAQLSTMDTATSSTSTGTISLQG
8536	KM389073	NAIESATGTTNGDTVSKNVCKGDSGTCGKNTSGTATTKISAVFTDEATLLSADTNNVSTSG
8536	KM389074	NAIESATGTTNGDTVSKNVCKGTGTSDTTNQCGTSTAGATTKISAVFTEDAAAQLSTMDTATSSTSTGTISLQG
8536	KM389075	NAIESATGTTSGDELSKKVCGKGTTSGNQCGVNATSGSTNNGKLSTVFSDEGTDAISSMETATISVQG
8536	KM389076	NAIESATGTTSGDELSKKVCGKGTTSGNQCGVNATSGSTNNGKLSTVFSDEGTDAISSMETATTSLQG
8536	KM389077	NAIESVTGTTNGQTVSQKVCGNGTGSSGSNCGKNTSGTAAQTNGKLSTVFSDEGTDAISSMETATISLQG
8536	KM389078	NAIESVTGTTNGQTVSQKVCGNGTTSGSNTNQCGVNAASGTTTKISAVFTDEAALLSADTNNVSTSG
8536	KM389079	TTIENTTGSGDELSKKVCGKGDRGTKCGVNATSGTAQTNGKLSTVFNTDGAEAISSMETATISVQG
8536	KM389080	TTIENTTGSGDELSKKVCGKGDSGTKCEVNATSGTAQTNGKLSTVFNTDGAEAISSMETATISVQG
8536	KM389081	TTIENTTGSGDELSKKVCGKGDSGTKCEVNATSGTAQTNGKLSTVFNTDGGEAISSMETATISVQG
8536	KM389082	TTIENTTGSGDELSKKVCGKGDSGTKCGVNATSGTAQTNGKLSTVFNTEGAEAISSMETATISVQG
8536	KM389083	TTIENTTGSGDELSKKVCGKGDSGTKCGVNATSGTAQTNGKLSTVFSDEGTDAISSMETATISVQG
8536	KM389084	TTIENTTGSGDVSQKVCKGDSGTCGKNTSGTAAQTNGKLSTVFSEEGTDAISSMETATISLQG
8536	KM389085	TTVEAATNGQTVSQKVCGNGTGSSGTQCGTSTAGATSGSSGTTQRKISEVFTSDTETAQLSTMENTSTTSGATIST
8536	KM389086	TTVEAATNGQTVSQKVCKGTGSTGTKCGTTDSTATTKISAVFTEGADAISSMDTTASGTSNTISFQG
8536	KM389087	TTVEAATNGQTVSQKVCKGTGSTGTKCGTTDSTATTKISAVFTEGADAIWSMDTTASGTSNTISLQG
4511	KM389088	ATIENTTGGGDVSQKVCKGTGTGGTNQCGKNTADGNATTKISAVFTEGTEAISSMETATSGATISTSG
4511	KM389089	ATIENTTGSGDVSQKVCGNGTSGTGGTQCGKNSGDTNGSSTTQHKISAVFTDEATLLSAAGDAINTTG
4511	KM389090	ATIENTTGSGDVSQKVCKGENNKCGVNATSGTTAQTKISAVFTSDTETAQLSTMDTATGSTGTIHLQG
4511	KM389091	ATIENTTGSGDVSQKVCKGTGTGGTNQCGKNTADGNATTKISAVFTEGTEAISSMETATFSLQG
4511	KM389092	ATIENTTGSGDVSQKVCKGTGTGGTNQCGKNTADGNATTQRKISEVFTSDTETAQLSSDTTNINTTG

4511	KM389093	ATIENTTGSGDVSQKVCKGTGTSGNQCGKNTADGNATTKISAVFTEGTEAISSMETATISLQG
4511	KM389094	KALEGVTGGDKVSQKVSNRENNKCGVNATSDTATTKISAVFTEGTDAISADTNNFSTSG
4511	KM389095	KAVEGTTNGEEVSQNVCGKGEGSNGTKKCGTTDSTATTQRKISEVFTSDTETAQLSSDTTNINTTG
4511	KM389096	KAVEGTTNGEKVSQNVCGKGEGSNGTKKCGTTDSTATTQRKISEVSTSDTETAQLSSDTTNINTTG
4511	KM389097	KAVEGVTGDDKVSQNVCKGDSGTKCGVNATSGTAQTKISAVFTEGTDTATQLSADTNNVSTSG
4511	KM389098	KAVEGVTGGDELSKKVCGKGTTSGNQCGVNATSGSTNNGKLSTVFNTDGAEAISSMDTTARGTSNTISLQG
4511	KM389099	KAVEGVTGGDELSKKVCGKGTTSGTGGTNQCGTGTAGATNNNGKITQAFTADSDTTLLSAESSTSNGTISVQG
4511	KM389100	KAVEGVTGGDELSKKVCKGENNKCGVNATSGSTTKISAVFTDEAALLSAAGDTINTTG
4511	KM389101	KAVEGVTGGDELSKKVCKGENNKCGVNATSGSTTKISAVFTDEATLLSAAGDTVNTTG
4511	KM389102	KAVEGVTGGDELSKKVCKGTGTGGTNQCGKNTADGNATTKISAVFTEGTEAISSMETATISLQG
4511	KM389103	KAVEGVTGGDELSKKVCKGTGTGSNTNQCGKNTADGNATTQRKISEVFTSDTETAQLSSDTTNINTTG
4511	KM389104	KAVEGVTGGDELSKKVCKGTGTGSNTNQCGTSTAGATATQHKISEVFTEGTDTATLLSADTNNVSTSG
4511	KM389105	KAVEGVTGGDELSKKVCKGTGTGSNTNQCGTSTAGATATQHKISEVFTEGTEAISSMETATISLQG
4511	KM389106	KAVEGVTGGDELSKKVCKGTGTSGNQCGKNTADGNATTKISAVFTEGTEAISSMETATISLQG
4511	KM389107	KAVEGVTGGDELSKKVCKGTTNQCGKNTADGTTAPTKISAVFTEDAAAQLSTMDNTTINTTG
4511	KM389108	KAVEGVTGGDELSKKVCKGTTNQCGKNTADGTTATTKISAVFTEDAAAQLSTMDNTTINTTG
4511	KM389109	KAVEGVTGGDELSKKVCRGENNKCGVNATSGSTTKISAVFTDEATLLSAAGNTINTTG
4511	KM389110	KAVEGVTGGDEPSKKVCKGENNKCGVNATSGSTTKISAVFTDEATLLSAAGDTINTTG
4511	KM389111	KAVEGVTGGDKVSQNACKGDSGTKCGVNATSGTAQTKISAVFTEGTDTATQLSADTNNVSTSG
4511	KM389112	KAVEGVTGGDKVSQNVCEGDSGAKCGVNAPSGTAQTKISAVFTEGTDSATQLSADTNNVSTSG
4511	KM389113	KAVEGVTGGDKVSQNVCKGDSGTKCGVNATSGTAQTKISAVFTDEATLLSAAGDTINTTG
4511	KM389114	KAVEGVTGGDKVSQNVCKGDSGTKCGVNATSGTAQTKISAVFTEGTDTATQLSADTNNVCTSG
4511	KM389115	KAVEGVTGGDKVSQNVCKGDSGTKCGVNATSGTAQTKISAVFTEGTDTATQLSADTNSVSTSG
4511	KM389116	KAVEGVTGGDKVSQNVCKGENNKCGVNATSGTATTKISAVFTEDAAAQLSTMDNTTINTTG
4511	KM389117	KAVENATNGDKVSQNVCGKGTGTGSDGNTKKCGTNDGTTATQRKISEVFTDEATLLSAAGDTINT
4511	KM389118	KAVESATGTTNGQTVSQNVCGKGTTSGSNTKCGVNATSGTAQTKISAVFTEGTDTATQLSADTNNVSTSG
4511	KM389119	KAVESATGTTNGQTVSQNVCGKGTTSGSNTNQCGVNATNGSSTTQHKISEVFTEGTEAATQLSADTNNVSTSG
4511	KM389120	KLVKCVTGGDKVSLNVCKGENNKCGVNATSGTATTEISVVFTEGTDVISVHTNNVGTSG
4511	KM389121	NAIESATGTTNGDTVSKNVCKGTGTSDTTNQCGTSTAGATTKISAVFTEDAAGQLSTMDTATSSTSTGTIGLQG
4511	KM389122	NAIESATGTTNGEKVSQKVCGNGTGSTGTTCGKSADSQSTNSKLGTVFNAEGADAISSMDTTTASGTSNTISLQG

4511	KM389123	NAIESATGTTNGEKVSQKVCGNGTSGTGGTQCGKNSGDTNGSSTTQHKISAVFTEDAAAQLSTTDNTTINTTG
4511	KM389124	NAIESATGTTNGEKVSQNVCKGDSGTKCGVNATSGTAQTKISAVFTEGTDTATQLSADTNNVSTSG
4511	KM389125	NAIESVTGTTNGQTVSQKVCGKGTTSGSNTNQCGKNTTDSTNNNGKITQAFTADSDTTLLSAESSNISTSG
4511	KM389126	NAIESVTGTTNGQTVSQKVCGKGTTSGSNTNQCGTSTAGATTKISAVFTEDAAAQLSTMDTATSSTSTGTISLQG
4511	KM389127	TTIENTTGSGDVSQKVCKGTGTGGTNQCGKNTADGTTKISAVFTSDTETAQLSSDTTNIDTTG
4511	KM389128	TTVEAATNGQTVSQKVCGKGTTSGTGGTNQCGTGTAGATTKISAVFTEGTDAISSMETATSGATISTSG
4511	KM389129	TTVEAATNGQTVSQKVCGNGTGSNCGVNSGTTGSTNGNKISAVFSAEGAEAISSMDTTSNGTTINVSG
4511	KM389130	TTVEAATNGQTVSQKVCGNGTSGGSTGTCGKNATSGSTNNNGKITQAFTADSDTTLLSAESSTSNGTISVQG
02V	KM389131	KAVENATGTTNGEKVSQKVCGTTSGSTNQCGKNTADGTTATTKISDAFTAGGAELLSSDTNKANIDTTG
02V	KM389132	NAIESVTGTTNGQTVSQKVCGKGTGSTGSSGNKCGTTDSTATTKISAVFTEDAAAQLSTMDNTTINTTG
68N	KM389133	KAVEGTTNGEKVSQNVCGNGTGSSGSNCGKNTSGTTATQRKISEVFTDEATLLSADTNNVSTSG
68N	KM389134	KAVEGVTGGDELSKKVCKGTGTGGTNQCGVNAASGTTTKISAVFTDEATLLSADTNNASTSG
68N	KM389135	KAVEGVTGGDELSKKVCKGTGTGSGSNCGKNTTDSTNNNGKITQAFTADSDTTLLSAAGDTINTTG
68N	KM389136	KAVENATGTTNGEKVSQKVCGTTSGSTNQCGTSTAGATNNGKLSTVFNTDGAEAISSMDTTINLQG
68N	KM389137	KAVENATGTTNGEKVSQNVCGNGTGSSGSNCGKNTSGTTATQRKISEVFTDEATLLSADTNNVSTSG
68N	KM389138	NAIESATGTTNGDKVSQNVCGKGTGTGGTNQCGTSTAGATNNGKLSTVFNTDGAEAISSMETATISLQG
68N	KM389139	NAIESATGTTNGEKVSQKVCGNGTGSSGSNHCGVNTGNTNKISAVFSAEGADAISSMDTTTTSGASGTISVQG
68N	KM389140	NAIESATGTTNGEKVSQKVCGNGTGSSGSNHCGVNTGNTNNGKLSTVFNTDGAEAISSMETATISLQG
68N	KM389141	NAIESATGTTNGEKVSQNVCGKGTGTGGTNQCGTSTAGATNNGKLSTVFNTDGAEAISSMETATISLQG
